# Precision Medicine: Personalizing Healthcare by Bridging Aging, Genetics, and Global Diversity

**DOI:** 10.3390/healthcare13131529

**Published:** 2025-06-26

**Authors:** Maria Edvardsson, Menikae K. Heenkenda

**Affiliations:** 1 Primary Health Care Center, 581 95 Linköping, Sweden; 2 Department of Biomedical and Clinical Sciences, Division of Clinical Chemistry and Pharmacology, Linköping University, 581 83 Linköping, Sweden

**Keywords:** precision medicine, aging, genetic diversity

## Abstract

Precision medicine transforms healthcare by tailoring prevention, diagnosis, and treatment strategies to individual characteristics such as genetics, molecular profiles, environmental factors, and lifestyle. This approach has shown promise in improving treatment efficacy, minimizing adverse effects, and enhancing disease prevention across various conditions, including age-related illnesses, cancer, type 2 diabetes, cardiovascular disease, and rare genetic disorders. However, major challenges remain that limit the potential of precision medicine. A key limitation is the underrepresentation of diverse populations in genetic research, leading to disparities in treatment outcomes and the potential misinterpretation of genetic risks. Current clinical reference intervals often fail to reflect the biological changes associated with aging, increasing the risk of misdiagnosis or inappropriate treatment in older adults. Our model calls for a broader, more inclusive framework, one that incorporates not only individual variability but also population-level factors such as aging and genetic diversity. Emerging technologies in artificial intelligence (AI), digital health, and multi-omics can help support this expanded approach. Precision medicine must include underrepresented populations in research, develop age-specific clinical guidelines, and address socioeconomic barriers. Here, we provide a brief introduction to our model. By integrating aging and genetics, precision medicine can evolve into a truly global approach—one that promotes health equity, respects biological diversity, and improves outcomes for all populations.

## 1. Introduction

Precision medicine transforms healthcare by customizing treatments to align individual characteristics, including genetic profiles, molecular biomarkers, environmental factors, and lifestyle choices [1]. Although the concept is well-established, this conceptual review explores the intersection of precision medicine, aging, and global genomic diversity, with a focus on advancing equity and representation. Rather than offering a systematic evidence review, the article synthesizes emerging themes to propose a framework for future research and policy. By examining how biological aging and population-specific genomic variation influence individualized care, the manuscript highlights opportunities for precision medicine to better serve aging populations and historically underrepresented groups. This tailored approach enhances treatment efficacy, reduces adverse effects, and provides a pathway to more effective disease prevention. As populations worldwide face the burden of major global health challenges such as cancer, type 2 diabetes, and cardiovascular diseases, precision medicine offers the potential to revolutionize their management. These diseases, which rank among the most common and deadly conditions globally, are influenced by complex interactions between aging processes, genetic predispositions, and lifestyle factors.

While numerous reviews discuss precision medicine, few emphasize the distinct vulnerabilities of older adults and the policy-level barriers that limit access to genomic tools in low-resource or non-European populations. This review aims to fill that gap.

Despite its transformative potential, precision medicine has yet to address the unique needs of aging populations fully. Older adults, who are disproportionately affected by chronic diseases, have been underrepresented in precision medicine research, resulting in a critical gap in understanding how age-related changes and frailty influence therapeutic outcomes. Frailty, a multifaceted condition characterized by declining physical and cognitive abilities, presents distinct challenges for healthcare interventions. Definitions of frailty, ranging from clinical syndromes to cumulative health deficits measured by frailty indices, underscore the heterogeneity of aging populations and the need for tailored healthcare solutions.

Statements like “healthcare systems have historically adopted a one-size-fits-all approach” have often described Euro–American clinical practices that relied on standardized diagnostic criteria and pharmacological responses, without adjusting for genetic ancestry or physiological differences linked to aging. For example, U.S. drug trials between 1990 and 2010 rarely included patients over 70 or those with multimorbidities, limiting their generalizability [2].

Group-based reference intervals are often used to compare changes in biomarkers for the person one wishes to examine. Requirements for inclusion in the reference population are to be healthy, free from diseases, and to take medication [3]. A consequence is that a large proportion of the total population is excluded, as with increasing age the risk of suffering from diseases and undergoing subsequent medication treatment increases [4,5]. Several attempts to create clinically useful reference intervals for individuals of different ages and with different disease burdens have been made, but these are complex problems and there is no consensus on the procedure [6,7,8,9,10]. Nor is there any consensus on how the health status of the elderly should be assessed [11,12,13].

Advances in genomics, AI, and digital health technologies have fueled the development of precision medicine, offering new avenues for targeted therapies. However, challenges remain, including the underrepresentation of diverse populations in genetic research and the need to update clinical reference intervals to address the unique needs of aging and resource-limited populations. Genetic variability, particularly population-specific differences in drug metabolism and disease susceptibility, further emphasizes the importance of global diversity in precision medicine strategies. There have been recent efforts that exemplify progress toward these goals. The NIH’s All of Us Research Program includes over 78% of participants from groups historically underrepresented in biomedical research and has prioritized aging-specific data structures [14]. Genomics England has developed a haplotype reference panel based on more than 78,000 individuals with increased imputation accuracy across age cohorts [15]. Similarly, Japan’s jMorp project provides multi-omics reference data stratified by age and sex, offering a template for age-aware genomics in non-Western populations [16].

This review builds on these advances by proposing that precision medicine must extend beyond individual tailoring to encompass demographic and policy dimensions. Specifically, we advocate for an approach that respects biological variability across the life course, ensures inclusive data infrastructures, and aligns with emerging global equity frameworks.

By addressing these dimensions, we aim to explore how precision medicine can provide equitable, effective, and individualized healthcare solutions, ensuring that older adults and diverse populations are no longer overlooked in the era of personalized healthcare.

## 2. Scope and Methodology of the Review

This review adopts a conceptual, narrative approach to synthesize current knowledge on the intersection of precision medicine, aging, genetics, and global health equity. We selected literature from both foundational and recent sources using targeted searches in PubMed, Scopus, and Google Scholar. Search terms included “precision medicine”, “aging”, “genomics”, “frailty”, “reference interval”, “health disparities”, and “equity in clinical research.” We prioritized peer-reviewed articles and policy documents relevant to adult and aging populations, including underrepresented groups. In addition to academic literature, we reviewed international reports and data from organizations such as the WHO and NIH.

## 3. The Impact of Aging on Health

This section outlines how aging affects biological systems and contributes to disease vulnerability. It also sets the foundation for understanding why precision medicine must adapt to age-specific needs.

### 3.1. Biological Changes

As an individual ages, the body undergoes numerous changes, many of which are influenced by various stressors and defense mechanisms. These stressors include physical factors (such as ultraviolet and gamma radiation), chemical agents (like oxygen free radicals and reduced glucose levels), and biological threats (including viruses and bacteria) [17]. In response, the body activates anti-stress responses aimed at maintaining homeostasis.

Age-related changes are often accompanied by a decline in organ function, including a loss of muscle mass due to malnutrition and the hormonal shifts associated with menopause and andropause. These shifts contribute to increased vulnerability to chronic conditions such as heart failure and stroke, which are linked to alterations in cardiac and vascular structure and function [18]. Similarly, renal function tends to deteriorate with age, primarily due to changes in the glomeruli, compounded by conditions like diabetes, heart failure, malignancy, and systemic inflammatory diseases [18].

In addition to these systemic changes, sensory losses, as well as difficulties with chewing and swallowing, can lead to reduced dietary intake, which further increases the risk of malnutrition [19]. This, in turn, leads to a heightened risk of morbidity, frequent hospital admissions, and increased dependency on daily activities, posing significant challenges for healthcare management and quality of life in older adults [20].

### 3.2. Immune Decline

As individuals age, significant changes occur in cellular processes and immune function. The relative number of apoptotic cells increases in individuals aged 80 years and older [21], contributing to a decline in the body’s ability to maintain healthy tissue. Additionally, immune system function deteriorates with age, with a notable decrease in peripheral blood T-cells and a slower, more transient antibody response to pathogens [22].

This immunosenescence plays a critical role in increasing vulnerability to acute infections, including influenza, pneumonia, urinary tract infections, and intra-abdominal sepsis [23,24,25]. To ensure these diagnoses, various protocols are used today—protocols and flow charts that lead to a proposal of actions. Protocols may include arterial blood gas, and blood tests to check levels of inflammatory biomarkers, liver status, and various enzymes. Urine analysis or the culture of urine or blood may also be relevant. Even if the laboratories deliver the outcome of the analyses as fast as possible, the time taken may, on some occasions, be too long and the outcome will be delivered too late. All of the outcomes of the analyses will be compared with group-based reference intervals. The clinical investigation may be quicker and more useful in some of these situations when there is not much time to wait for the results from the laboratories or when the reference intervals are not appropriate for the examined individual. An investigation including temperature, appetite, malaise, fatigue, an individual “not behaving as usual,” etc. could be appropriate in some cases.

Furthermore, chronic low-grade inflammation, often associated with aging, is increasingly recognized as a driving factor not only in the aging process itself but also in the development of age-related diseases such as cancer, Alzheimer’s disease, myocardial infarction, stroke, and type 2 diabetes [26,27,28].

### 3.3. Disease-Specific Precision Medicine

Precision approaches must adapt to how aging alters disease expression and treatment responses. Below, we review applications in three major disease categories.

#### 3.3.1. Cancer

Cancer remains one of the leading causes of death worldwide, with nearly 10 million deaths in 2020. The most common cancers, breast, lung, colorectal, and prostate, share a complex interplay of genetic and environmental risk factors [29]. In flammation is a leading hallmark of the development and progression of cancer [30]. Strong connections have been identified between inflammation and tumorigenesis, including proliferation, invasion, angiogenesis, and metastasis [31]. More importantly, inflammation, mediated by cytokine release orchestrated by various cells within the tumor microenvironment, plays a critical role in these processes [32]. Beyond inflammation, genetic predisposition significantly affects cancer susceptibility. For example, BRCA1 and BRCA2 mutations increase breast and ovarian cancer risk, while TP53 mutations contribute to multiple cancer types [33,34]. Understanding these genetic variations is crucial for precision medicine, which tailors treatment based on a patient’s unique molecular profile.

#### 3.3.2. Type 2 Diabetes

Insulin resistance (IR) constitutes a significant health hazard on a global aspect. IR, which is closely related to metabolic syndrome (MetS), has been associated with the so-called Western lifestyle, characterized by high-calorie food consumption, limited physical activity, and excessive stress [35]. IR has been extensively linked to chronic low-grade inflammation and the production of proinflammatory cytokines, such as tumor necrosis factor-a (TNF-a), interleukin (IL)-6, IL-8, plasminogen activator inhibitor-1 (PAI-1), and monocyte chemoattractant protein-1 (MCP-1); their increased production is accompanied by elevated levels of C-reactive protein (CRP), a widely used inflammatory biomarker [36,37,38].

Platelet function investigation in affected individuals revealed that the density distribution of circulating platelets is altered in cases of Type 2 diabetes [39]. The platelets are probably affected by impaired glycemic control, but such pathophysiological relationships have been poorly investigated. After agonist stimulation in vitro, the degree of mitochondrial injury was higher in a group of elderly individuals with high HbA1c, mean 67 ± 10 mmol/L, compared with those with lower HbA1c, mean 49 ± 6 mmol/L [39].

#### 3.3.3. Cardiovascular Disease

As the leading cause of death, cardiovascular disease (CVD) and its associated treatment costs, will continue to increase [40]. By 2030, about 20% of the population will be aged 65 years and older, and CVD will result in 40% of all deaths. Aging has a high impact on the heart and the arterial system leading to an increase in CVD, including atherosclerosis, hypertension, myocardial infarction, and stroke. Examples of pathological alterations due to aging in cardiovascular tissue are hypertrophy, altered left ventricular (LV) diastolic function, diminished LV systolic reverse capacity, increased arterial thickening and stiffness, and impaired endothelial function [41]. Each system greatly affects the other. Age further affects heart rate modulation, with a decrease in both rate variability and maximum heart rate. Examination of the heart with electrocardiography (ECG) must become mandatory during health check-ups to make it possible to detect cardiovascular disease at an early stage and to prevent the prognosis above from coming true. Certainly, laboratory assessments like the investigation of levels of Troponin could give valuable information about the heart as well.

### 3.4. Different Stages of Aging

Older people with different diseases, but who were well treated with medications and not suffering from the disease, were in some studies called “pre-frail” [42,43]. The SUPER study used a multi-site, single-blind randomized controlled trial involving almost 2700 community-dwelling older adults in New Zealand [42]. They aimed to reduce physical frailty in the “pre-frail” by a complex intervention combining nutritional and physical activity interventions. However, no clear evidence of long-term impact was found after a two-year follow-up. In another multicenter randomized controlled study of 278 older people, group exercise programs were used [43]. After one year, the programs had positive effects on falling and physical performance in the “pre-frail”, but not in “frail” individuals. From our point of view, this definition is a little confusing, as “pre-frail” implies a state of being prior to becoming “frail”, but it is not certain that the person will ever be classified as “frail”. To more clearly depict the condition, we adopted the expression “moderately healthy” [44,45], which has been used by others [46,47]. We used two different definitions of “moderately healthy” in our investigations. One definition classified a person as moderately healthy if they had an Activities of Daily Living (ADL) score greater than a point, or a Mini-Mental State Examination (MMSE) score between 1 and 26, and some form of chronic disease or diseases [48,49,50]. In our study, we divided 568 older people into three groups: “healthy,” “moderately healthy,” and “frail”. We used impaired physical and cognitive ability to distinguish those we assessed as frail [6,7]. The reference population consisted of about 3000 healthy people from the Nordic countries, NORIP [51]. The outcomes indicated that all older people in the “healthy” and “moderately healthy” groups had similar levels to the reference population. The reason why the “moderately healthy” group was similar to the reference population might be that they were well treated, and because of that they had levels of biomarkers similar to those of the “healthy”. The “frail” group had lower levels of some biomarkers (albumin and alanine aminotransferase (ALT)) and higher levels of others (aspartate aminotransferase (AST) and gamma-glutamyl transferase (γ-GT) [44]. To some extent, these outcomes are supported by others [8,10,13,46,47]. The results suggest that levels of biomarkers in older people can be compared to the commonly used reference intervals, even for those over 65 years, if they are well treated for existing diseases.

Subsequently, we randomized 568 people, aged 80 or over, into two groups [45]. One of the groups was divided into “healthy”, “moderately healthy”, and “frail” categories based on individuals’ physical and cognitive abilities. The other group was divided into “healthy”, “moderately healthy”, and “frail” categories based on the frailty index (FI). This instrument includes variables that are available for the investigated person, such as function, cognition, comorbidity, and physical performance. We investigated whether there were differences in levels of commonly used biomarkers, depending on whether the person was assessed as “healthy”, “moderately healthy”, or “frail”, classified in different ways. The outcomes indicated that both groups of “healthy” and “moderately healthy” individuals were quite similar to the current reference intervals. But for the two groups of “frail” individuals, differences appeared. People classified as “frail” based on the FI showed significantly lower ALT, creatinine, and γ-GT levels compared to those classified as “frail” based on physical and cognitive abilities [45].

Frailty is a condition that describes when a person’s physical and cognitive abilities decline. In figurative language, the following expressions are often used: “Frailty is a complex, multifaceted concept” or “Frailty indicates something that is failing or faulty” [52]. Several attempts have been made to define frailty. Frailty can be defined as a clinical syndrome in which three or more of the following criteria are present: unintentional weight loss, self-reported exhaustion, weakness (grip strength), slow walking speed, and low physical activity [12]. Others suggest estimating the ability to perform daily activities together with cognitive abilities. Further, another method used by several researchers is to count deficits for a specific person, contributing to the person’s risk of death, that have occurred and in a form that creates an index, called the frailty index (FI) [13,48]. Conditions included are those known to be negative for the person, such as medical conditions, a failure to manage daily functions, impaired physical and cognitive abilities, and comorbidity. With current advancements in medication, it is not uncommon for people with chronic diseases to be well treated with appropriate medications such that they may not suffer from their illnesses or struggle to manage daily life independently.

It has been shown that individuals with rapid frailty processes and with high levels of trajectory may experience a greater risk of adverse outcome events including hospitalization and mortality [53]. This makes it important to identify older individuals at risk to prevent and slow down the frailty process. In a Chinese study (CHARLS), researchers identified three frailty trajectory groups, i.e., low-increase, moderate-increase, and high-increase trajectory. Individuals with unfavorable childhood and adulthood socioeconomic status were more likely to be in a high-increase frailty trajectory group. They stated that more attention should be paid to people with low socioeconomic status to reduce health inequalities in later life and slow down or reverse the progress of frailty [54].

As people age, they tend to use more prescription medications. Data from Europe show that while 46 percent of adults aged 45 to 54 use prescribed drugs, this rises to over 87 percent in those aged 75 and older [55]. Yet despite this high level of use, older adults, especially those who are frail or living with multiple health conditions, are still often excluded from clinical trials. This is due to factors such as upper age limits, comorbidities, or cognitive impairment [56,57]. Although the ICH E7 guidelines have long recommended the inclusion of older adults in drug studies, many trials still fall short in this respect [58]. Even when older participants are included, trials often focus on outcomes like survival rather than quality of life, independence, or physical function—factors that are especially important in this age group. To ensure that medications are safe and effective for those most likely to use them, clinical research needs to better reflect the realities of aging.

## 4. Reference Intervals

For many years, there have been carefully established rules for how reference intervals in laboratory medicine should be developed. Networks such as the International Federation of Clinical Chemistry (IFCC) work worldwide to promote a common view of how this approach should be.

### 4.1. Group-Based Reference Intervals

Group-based reference intervals are often used to compare changes in biomarkers for the person one wishes to examine. Producing reference intervals for a general population is a major challenge. It requires selecting the appropriate reference population, recruiting people who represent relevant demographic groups that meet the inclusion criteria, collecting, processing, and testing specimens, and finally, calculating reference values with the possible stratification of the data into subgroups. The procedure is carefully specified and is used worldwide [3,59,60].

One way to produce the reference interval is to use the Gaussian theory, by calculating the mean value and ±2 standard deviations (SD) [61]. The Gaussian theory is based on the occurrence of what is being investigated as normally distributed, which is not often the case with biological materials. Galen and Gambino [4] suggested using percentiles and that the reference interval should be between the 2.5th and the 97.5th percentiles, which is a common convention today [5,62]. Others have tried to solve this problem by using square root or log transformation [6,61] routines, which resulted in the inclusion of young and middle-aged people, who had not yet suffered from any chronic diseases and were not being treated with any medications [51]. In clinical work, men often represent the dominant part of the reference population. The reason for this is that blood donors and military servicemen are often included, as blood sampling is part of their roles.

### 4.2. Clinical Challenges

A consequence of using only people who have not been treated with disease drugs when developing reference values is that a large part of the total population is excluded since the risk of developing diseases, and undergoing subsequent drug treatment, increases with increasing age [18,19]. Hence, the outcomes of biomarker analysis in an older person with chronic diseases will be compared with group-based reference intervals derived from healthy, significantly younger people. This may result in misinterpretation when the outcomes from older people fall outside the reference interval, even if they would be at a suitable level for aged individuals [44,45,63]. This could result in misleading or even dangerous assessments. When an outcome falls outside the reference interval, the outcome is presented in the computer system in a red color or marked in another way to get the attention of the physician. In some cases, the expected value for an older person is lower or higher than for younger individuals; the value of the older person will be noticed through the red indicator in the computer system, as it falls outside the reference interval. In that case, the physician’s attention is called completely unnecessarily. In an even worse case, when the outcome is expected to be outside the current reference interval but falls within the interval because of old age or chronic disease, the outcome may not receive the necessary attention [44,45,63]. One cohort of older individuals in nursing homes was investigated during 2000–2001 where it was found that only nine out of one hundred thirty-eight individuals were without any disease [63]. The results therefore indicate that for 94% of the cohort, reference intervals could not be used when interpreting laboratory outcomes.

## 5. Why Does Genetics Matter in Precision Medicine?

Diagnostic genetic testing has a long history, dating back to prenatal diagnostics for hemoglobinopathies in the late 20th century. Landmark discoveries, such as identifying the Factor V Leiden (FVL) mutation in 1995, exemplify how genetics has shaped our understanding of thrombotic disorders. Recent advances in next-generation sequencing (NGS) and genome-wide association studies (GWAS) have further accelerated personalized medicine. These technologies enable the identification of single nucleotide variants (SNVs) associated with clinical phenotypes and allow whole exome or genome sequencing at unprecedented speed and affordability [64].

### 5.1. Global Genetic Diversity and Clinical Gaps

DNA recombination, migration, admixture, and natural selection shape human genetic diversity. Our genomes reflect this history, with variations between populations playing an important role in health and disease. While all humans share a common African origin and have more genetic similarities than differences, the existing variation is critical for understanding how different populations experience disease. Unfortunately, most large genetic studies, such as GWAS, have focused overwhelmingly on individuals of European descent. Despite representing only 16% of the global population, Europeans account for nearly 80% of participants in these studies. This imbalance limits the global applicability of genetic discoveries and poses challenges to advancing equitable healthcare, particularly in the field of precision medicine [65,66,67].

Populations across the world have unique genetic differences because of their histories, environments, and migrations. African populations, for example, have the greatest genetic diversity of any group, but they are one of the most underrepresented in genetic studies. This lack of research can lead to serious gaps in understanding diseases and treatments. For example, the TTR V122I mutation, which significantly increases the risk of a heart condition called amyloid cardiomyopathy, is common in people of African descent but rare in Europeans [68]. Without studying these populations, we might never discover such critical genetic factors [65,67].

Another issue is that genetic findings from European populations often do not work well for other groups. For example, genetic risk scores (GRS) are used to estimate the likelihood of developing certain diseases based on genetic data [69]. However, because genetic structures like linkage disequilibrium (LD) and allele frequencies vary between populations, these scores often fail to accurately predict disease risks in non-European groups. This makes it hard to apply precision medicine fairly and effectively [65,70].

### 5.2. Ancestry-Informed Risk and Pharmacogenomics

Thrombosis, a multifactorial disease influenced by genetic and environmental factors, exemplifies the complexity of personalized medicine. In venous thromboembolism (VTE), management strategies depend on whether the condition is provoked (e.g., surgery, immobility) or unprovoked. Long-term anticoagulation is typically reserved for unprovoked cases due to the higher recurrence risk, though predictive models such as the DASH score and Vienna model integrate clinical and biomarker data to guide therapy [71,72,73]. Genetic testing plays a selective role in thrombosis management. However, genetic variations influencing thrombotic risk differ across populations, underscoring the need for ancestry-informed approaches. However, recent findings indicate that thrombotic risk is not uniform across populations, as genetic variants like the PAR4 rs773902 SNP exhibit substantial differences in frequency depending on geographic ancestry. For example, while this variant has been associated with heightened platelet activation and an increased risk of thrombosis, its distribution varies significantly between East and West African populations, challenging the assumption that broad racial classifications can adequately capture genetic risk. Recognizing these differences is essential for refining genetic screening guidelines, optimizing anticoagulant therapy, and advancing precision medicine approaches tailored to diverse populations [74].

One of the most profound impacts of genetics in precision medicine is seen in pharmacogenomics, where genetic differences affect how individuals metabolize and respond to medications. For example, variations in the CYP2C9 and VKORC1 genes influence warfarin metabolism, requiring dosage adjustments to avoid complications such as bleeding or thrombosis [75,76,77]. Similarly, genetic variations in P2Y12 receptors affect the efficacy of antiplatelet therapy, demonstrating the necessity of integrating genetic testing into treatment plans [64]. Understanding genetic diversity is also vital for drug treatments. A good example of this is the antiretroviral drug efavirenz, used for treating HIV. Variants in the CYP2B6 gene, which are more common in African populations, can cause higher drug concentrations in the blood, leading to more side effects [78]. Similarly, G6PD deficiency, which is prevalent in some African and Mediterranean populations, can lead to dangerous reactions to certain medications [79]. Without studying these genetic differences, people from these populations may receive less effective or even harmful treatments [65,67]. These findings have significantly improved drug safety and efficacy by minimizing adverse reactions and optimizing therapeutic outcomes.

Studies that include diverse populations have already shown how valuable they can be. For instance, genetic research involving multiethnic groups has found new variants linked to diseases like asthma and type 2 diabetes that would not have been discovered in single-population studies [80,81]. Additionally, African-specific APOL1 variants have been linked to kidney disease, helping to explain some health disparities and pave the way for better treatments [82,83].

### 5.3. Genetic Data in Aging Populations

Genetic markers such as APOE4, known for its association with Alzheimer’s disease, and inflammatory pathway variants (e.g., IL-6) can help stratify risk in older adults [84,85,86]. Polygenic risk scores (PRSs) are increasingly used to predict susceptibility to chronic conditions such as type 2 diabetes, atrial fibrillation, and metabolic syndrome, enabling earlier preventive measures [69,87]. Yet few studies have validated PRSs specifically in aging or frail populations.

Recent studies suggest that combining different types of biological data, such as genetic, epigenetic, and protein-based information, with standard clinical markers can improve how we assess health risks and disease progression in older adults. This approach, often referred to as multi-omics, helps capture the complexity of aging and provides a more complete picture of an individual’s health [88]. At the same time, new digital health tools, such as wearable sensors and algorithms that measure frailty, are being used to monitor physical changes as they happen. These technologies make it possible to provide more timely and personalized care, especially for older adults whose health status may change quickly [89].

While genetic tools such as PRSs and pharmacogenomics are increasingly used to personalize healthcare, it is important to acknowledge that the influence of genetics may diminish with age relative to other factors. In older adults, the cumulative effects of lifestyle, environmental exposures, and comorbidities often play a more dominant role in disease development and outcomes [90,91].

This perspective does not diminish the value of genomics in aging populations but rather situates it as one element of a broader precision health strategy. A more effective approach will integrate genetic data with frailty indices, functional biomarkers, and behavioral health assessments to support individualized care in later life [92,93].

## 6. Toward a More Inclusive Precision Medicine Framework

Precision medicine has the potential to revolutionize healthcare by tailoring prevention, diagnosis, and treatment strategies based on individual variability in genes, environment, and lifestyle. However, realizing this potential requires addressing key challenges, particularly the underrepresentation of aging populations and ethnically diverse groups in genomic research. These gaps limit the generalizability of findings and may unintentionally exacerbate existing health disparities.

### 6.1. Closing the Gaps with Global Genomic Initiatives

Large-scale genomic studies have historically focused on populations of European descent, which limits the accuracy and effectiveness of genomic tools in other groups. According to Sirugo et al. (2019) [67], over 80% of GWAS participants were of European ancestry as of 2016, despite Europeans representing less than 20% of the global population. This imbalance has resulted in PRSs that underperform in non-European populations, often leading to inaccurate disease predictions and risk stratification [65,66,67].

In recent years, several initiatives have begun to address this gap. The All of Us Research Program in the United States aims to enroll one million participants, with a focus on communities historically underrepresented in biomedical research. By 2022, over 500,000 participants had been enrolled, with more than 77% identifying with underrepresented racial or ethnic groups, and over 50% aged 50 or older [14]. This approach strengthens the inclusiveness and applicability of precision medicine. The Genomics England program has also made significant progress. In 2024, a comprehensive haplotype reference panel covering more than 78,000 genomes was released, significantly improving imputation quality for rare variants and ancestrally diverse individuals [15]. Similarly, Japan’s jMorp project offers population-specific, age-stratified multi-omics data, enabling precision healthcare grounded in national demographic and biological diversity [16].

Global equity also depends on integrating genomic diversity into clinical practice and infrastructure. Biobank initiatives such as GenomeAsia 100K, China Kadoorie Biobank, and the H3Africa Consortium contribute valuable data from Asian and African populations that can improve PRS calibration and disease modeling across diverse ancestry groups [94,95,96]. In addition, the growing role of AI and machine learning enables researchers to integrate complex genomic, clinical, and environmental data. For instance, Martin et al. (2019) showed that ancestry-specific training improves PRS performance across diverse groups [97]. However, the equitable deployment of AI requires high-quality, diverse input data, without which algorithms risk replicating existing biases.

Equity also requires ethical, community-based approaches. Historical mistrust in research among marginalized groups, due to unethical practices such as the Tuskegee Study, has had long-term consequences [98]. As such, community engagement, transparent informed consent, and culturally sensitive recruitment strategies are essential. Frameworks from organizations like the WHO emphasize fairness, reciprocity, and benefit-sharing in global genomic science [29].

### 6.2. Proposed Model for Individualized and Age-Adapted Health Monitoring

If we want to expand the use of precision medicine in healthcare, we need to change the way we work. Today, it can be difficult, inaccessible, and expensive to examine one’s own health, as our healthcare focuses on helping us when illness has already occurred. Healthcare professionals do not have the time or financial resources to examine healthy individuals. For the individual, and also from an economic perspective, it would probably be more profitable to examine the person before they develop a chronic disease and, if so, to find the chronic disease at an early stage. Appropriate examinations should be made available to prevent the disease from worsening and thus prevent higher healthcare costs, but also to promote the quality of life of the individual.

For moderately healthy individuals with well-treated chronic conditions, annual follow-up of disease progression, which is commonly used today, is an appropriate time interval. Of course, the person in question should contact their healthcare provider if anything should arise between check-ups.

When investigating frail individuals, different routines are needed. First and foremost, consensus is needed on how to know whether a person is defined as frail or not. Because comparisons of laboratory test results and other measurements with healthy and younger individuals can be misleading, continuous monitoring and individualized intervals are needed.

## 7. Conclusions

Precision medicine can only succeed if it includes everyone, not just a select few. To make this vision a reality, it is essential to incorporate genetic diversity, consider the biological realities of aging, and design systems that work in everyday clinical settings. Initiatives like All of Us in the United States, Genomics England, and Japan’s jMorp project are already showing how this can be done in practice.

**Figure 1:**
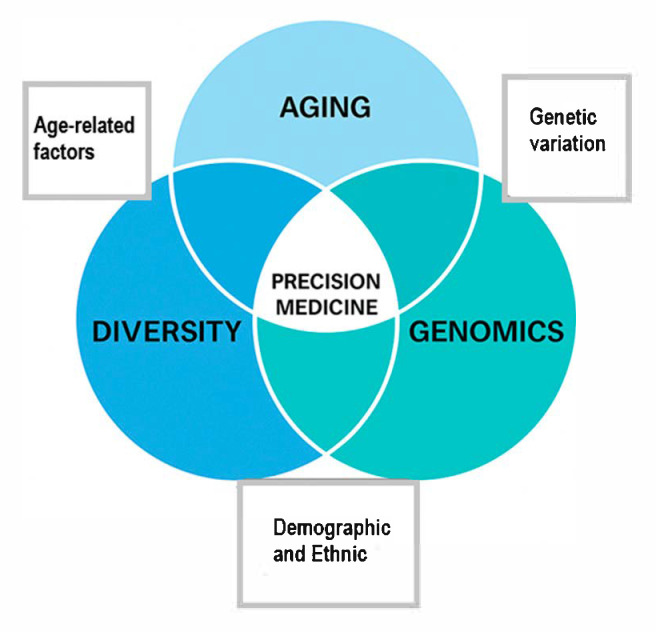
Conceptual framework for inclusive precision medicine. This diagram shows how precision medicine sits at the intersection of three essential areas: aging, genomics, and diversity. Each of these elements contributes to unique challenges and opportunities. Aging brings biological changes and increased vulnerability to disease. Genomics adds insight into individual risk based on genetic variation. Diversity reflects demographic and ethnic factors that influence access, diagnosis, and treatment. At the center, where these areas overlap, is a vision for more inclusive and effective precision healthcare. This framework highlights the importance of developing approaches that are not only scientifically advanced but also equitable, age conscious, and relevant across global populations.

Figure 1 presents a simple framework that brings together three critical elements: aging, genomics, and equity. These areas must be addressed together to create a truly global and inclusive approach to precision medicine. Moving forward, strong partnerships between researchers, policymakers, and healthcare professionals will be needed to build healthcare systems that are fair, practical, and prepared for the future.
